# Biology of the BKPyV: An Update

**DOI:** 10.3390/v9110327

**Published:** 2017-11-03

**Authors:** Francois Helle, Etienne Brochot, Lynda Handala, Elodie Martin, Sandrine Castelain, Catherine Francois, Gilles Duverlie

**Affiliations:** EA4294, Unité de Virologie Clinique et Fondamentale, Centre Universitaire de Recherche en Santé, Centre Hospitalier Universitaire et Université de Picardie Jules Verne, 80054 Amiens, France; etienne.brochot@u-picardie.fr (E.B.); l.handala@gmail.com (L.H.); e.martin2711@gmail.com (E.M.); sandrine.castelain@u-picardie.fr (S.C.); catherine.francois@u-picardie.fr (C.F.); gilles.duverlie@u-picardie.fr (G.D.)

**Keywords:** polyomavirus, noncoding control region, archetype, rearranged form, TAg, VP1, Agno, ganglioside, ERAD

## Abstract

The BK virus (BKPyV) is a member of the *Polyomaviridae* family first isolated in 1971. BKPyV causes frequent infections during childhood and establishes persistent infections with minimal clinical implications within renal tubular cells and the urothelium. However, reactivation of BKPyV in immunocompromised individuals may cause serious complications. In particular, with the implementation of more potent immunosuppressive drugs in the last decade, BKPyV has become an emerging pathogen in kidney and bone marrow transplant recipients where it often causes associated nephropathy and haemorrhagic cystitis, respectively. Unfortunately, no specific antiviral against BKPyV has been approved yet and the only therapeutic option is a modulation of the immunosuppressive drug regimen to improve immune control though it may increase the risk of rejection. A better understanding of the BKPyV life cycle is thus needed to develop efficient treatment against this virus. In this review, we provide an update on recent advances in understanding the biology of BKPyV.

## 1. Introduction

The BK virus (BKPyV) was first isolated in 1971, by Gardner et al., after inoculation of Vero cells with urine samples from a 39-year-old Sudanese male with the initials B.K., who had undergone renal transplantation because of chronic pyelonephritis and advanced renal failure [1]. In a recent revision of the virus taxonomy, this virus has been classified in the *Betapolyomavirus* genus of the *Polyomaviridae* family [2]. It shares a 75% and 69% sequence homology with JCPyV and SV40, respectively [3]. Genetic heterogeneity has been used to divide BKPyV into four major subtypes, I, II, III and IV, further divided into subgroups [4]. Subtype I, the most prevalent, is distributed worldwide, subtype IV in East Asia and Europe and subtypes II and III are rarely observed [5]. In addition, it has been shown that subtypes II, III and IV and subgroups Ib1 and Ib2, behave as five fully distinct serotypes [6]. Primary infections likely occur in early childhood and serum antibodies can be detected in more than 80% of the individuals by the age of 21, suggesting that the majority of the world’s adult population is latently infected [7]. Serological association of primary infection with upper respiratory infection suggests that transmission most likely occurs through a fecal-oral or a respiratory route but BKPyV may also be transmitted via semen, transfusion or organ transplantation, particularly renal allografts [8]. It is generally assumed that the initial infection occurs in the tonsils, with infected monocytes spreading the virus to other tissues and organs, particularly the kidney. The primary infection appears to be asymptomatic. Then, the virus persists in epithelial cells of the kidney and the urogenital tract in a true state of viral latency with minimal episomal replication. However, the virus can be reactivated and induce diverse complications, typically during immunosuppressive disease or treatment. In particular, with the use of more efficient immunosuppressive therapies, such as tacrolimus and mycophenolate mofetil, BKPyV became a major issue for bone marrow and renal transplant patients since it often causes haemorrhagic cystitis and polyomavirus nephropathy, respectively. Reactivation of latent BKPyV infection progresses to polyomavirus-associated nephropathy in about 5% of kidney recipients, which results in loss of graft function in 50–70% of these patients. Unfortunately, no specific antiviral against BKPyV has been approved yet and the only therapeutic option in BKPyV nephritis is a modulation of the immunosuppressive drug regimen to improve immune control and reduce BKPyV disease though it may increase the inherent risk of rejection.

The aim of this review is to present a summary of the accrued knowledge about BKPyV biology. We summarize the information regarding the BKPyV particle, the viral genome and proteins as well as the model systems for BKPyV research. We also review the data concerning the BKPyV life cycle. Clinical aspects, immune regulation and emerging therapies related to BKPyV have been reviewed in recent publications [9,10] and thus will not be addressed here.

## 2. The BKPyV Particle

**Figure 1:**
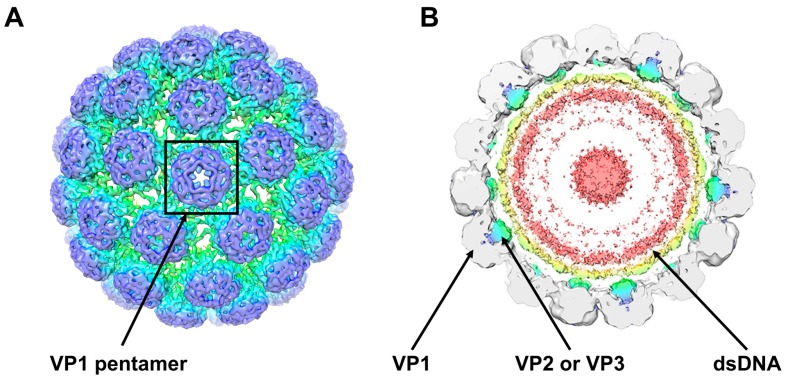
Cryo-electron microscopy structure of BK virus (BKPyV) viral particles (Adapted from [11]). (**A**) External view of the BKPyV virion shown at a contour level of 0.022. A viral protein VP1 pentamer is highlighted; (**B**) View of a 40-Å-thick slab through the unsharpened/unmasked virion map shown at a contour level of 0.0034. Pyramidal density below each VP1 penton and two shells of electron density adjacent to the inner capsid layer can be seen. The density within 6 Å of the fitted coordinates for SV40 VP1 is coloured grey. Density for VP2 and VP3 is coloured blue/green and for packaged double stranded DNA (dsDNA) yellow/pink.

The subnanometer resolution structure of native infectious BKPyV virions (Figure 1), obtained by cryo-electron microscopy, has recently been presented [11]. BKPyV infectious viral particles are non-enveloped, with a diameter of ~45 nm [11,12] and a density of 1.34 g/mL [13]. These particles enclose the viral genome that is packed with host cell histones (H2A, H2B, H3 and H4) [13,14]. On average, each genome contains about 20 nucleosomes [15]. The viral protein VP1 is the major protein of virions, accounting for 80% of their total protein content. Indeed, BKPyV capsids are composed of 360 molecules of VP1 forming 72 protruding pentamers arranged in a T = 7d icosahedral structure [11,12,16,17], stabilized by intra- and inter-pentameric disulphide bonds as well as Ca^2+^ cations [18]. In addition to these pentamers residing at the outer surface of the viral capsid, two other viral proteins, VP2 and VP3, reside in the inner part of the particles (Figure 1). The protruding C-arms of the major capsid protein VP1 allow flexible interaction with the neighbouring pentamers [11,17], whereas a single copy of the C-terminus of either VP2 or VP3 is able to form a hairpin-like structure that is inserted into the cavity of the VP1 pentamers [19]. Sixty of the 72 pentamers are coordinated with six adjacent pentamers and the other twelve pentamers are located on the icosahedral vertices of the capsid, coordinated with five surrounding pentamers [11,20].

## 3. The BKPyV Genome

**Figure 2:**
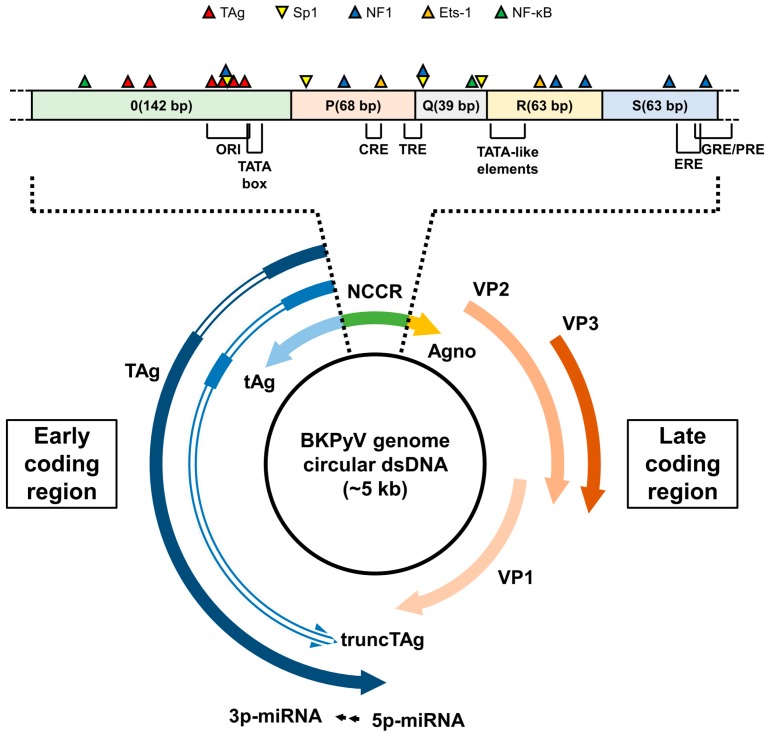
Genome map of BKPyV. (**Bottom**) The BKPyV genome is a closed circular, double-stranded DNA molecule of approximately 5 kb. The transcription of early and late coding regions proceeds in a bidirectional way from the origin of replication (ORI) that is located within the noncoding control region (NCCR). The early coding region encodes large tumour antigen (Tag), small tumour antigen (tAg) and truncated TAg (truncTAg) that are produced from different alternatively spliced mRNAs. Introns in the early coding region are represented by double lines. The late coding region encodes the structural proteins VP1, VP2 and VP3 as well as the Agno protein. These proteins are translated from two classes of late RNAs, 16S and 19S, that are generated by alternative splicing from a common pre-mRNA. The 19S RNA is translated to yield both VP2 and VP3 while the 16S RNA species is translated to yield Agno and VP1. The BKPyV genome also encodes two miRNAs, 5p-miRNA and 3p-miRNA, produced after processing of a common pre-miRNA hairpin and perfectly complementary to the TAg encoding mRNAs. (**Top**) The schematic organization of the BKPyV archetype non-coding control region (NCCR) is represented. It is divided into five sequence blocks (O, P, Q, R and S). It includes the origin of replication (ORI), TATA box and TATA-like elements. The positions of different sites important for the binding of TAg and the transcription factors Sp1, NF1, Ets-1 and nuclear factor κB (NF-κB), as well as cAMP-, phorbol ester-, glucocorticoid/progesterone- and oestrogen responsive-elements (CRE, TRE, GRE/PRE and ERE, respectively) are also mentioned. CRE: cAMP responsive-element; TRE: phorbol ester responsive-element; GRE/PRE: glucocorticoid/progesterone responsive-element; ERE: oestrogen responsive-element.

The BKPyV genome is a closed circular double-stranded DNA molecule of approximately 5 kb (3.4 × 10^6^ daltons) that replicates bidirectionally from a unique origin (Figure 2). It is composed of two highly conserved regions coding for early and late proteins respectively, separated by a non-coding control region (NCCR) of approximately 400 bp. The early genes encode the large tumour antigen (TAg), the small tumour antigens (tAg) and the truncated TAg (truncTAg) that are expressed by alternatively spliced mRNAs soon after infection of the host cell. The late genes, encoding the structural proteins VP1, VP2 and VP3 as well as the Agno protein, are expressed after genomic replication has been initiated. These proteins are translated from two classes of late RNAs, 16S and 19S, that are generated by alternative splicing from a common pre-mRNA. The 19S RNA is translated to yield both VP2 and VP3 while the 16S RNA species is translated to yield VP1 and Agno.

The BKPyV strains that predominate in the urine are called “archetypal strains” and are thought to be the transmissible form of the virus circulating in the human population [21,22]. The NCCR of BKPyV archetypes (the prototype of which is the WW strain [21]) is divided into five sequence blocks called, starting from the early side, O(142 bp), which includes the origin of replication as well as a TATA-box and P(68 bp)-Q(39 bp)-R(63 bp)-S(63 bp) that contain TATA-like elements and the regulatory regions for early and late genes expression (Figure 2) [3,23,24]. Indeed, approximately 30 transcription factor binding sites are consistently predicted in silico [23,25,26]. Recently, Bethge et al. extensively studied the contribution of several transcription factor binding sites and evidenced the major role played by Sp1 [23,24]. They demonstrated that the bidirectional balance of early and late viral gene region expression is dependent on affinity, strand orientation and the number of binding sites for this transcription factor. The additional role played by other transcription factors such as NF1, Ets-1 or NF-κB was evidenced in several studies [23,27]. Furthermore, Moens et al. identified cAMP-, phorbol ester-, glucocorticoid/progesterone- and oestrogen-responsive-elements that confer regulation of transcription and replication [28,29].

However, while most viral strains reveal a strong sequence conservation in the protein coding regions, the NCCR exhibits considerable variation between different BKPyV isolates. As a result, rearranged forms of the NCCR are commonly found in the kidney and other tissues, often in association with disease [3]. Rearrangement of most naturally occurring NCCRs involves duplication or triplication of the P region, including portions of the neighbouring O and Q regions. In addition, deletions are found to occur anywhere within the P, Q, R, or S regions but frequently the deletion includes all or part of the R region. Nonetheless, there seems to be a selection to preserve the P block as all described variants so far retain P sequences. Most known variants have also retained the S block, indicating the importance of these sequences [30]. Importantly, duplications, deletions and new junctions of the P, Q, R and S sequence blocks result in the creation and deletion of transcription factors binding sites [23,31]. Besides, different studies demonstrated that archetypal NCCRs conferred a strong late gene expression and a relatively weak early gene expression contrary to rearranged NCCRs that showed increased early gene expression and decreased late gene expression, irrespective of insertion or deletion architectures [31,32]. Several studies have shown that the anatomy of the NCCR also determines the host cell tropism and permissivity, as well as the oncogenic potential in vitro [26]. Thus, the NCCR heterogeneity likely reflects the ability of polyomaviruses to adapt to new cellular environments and the NCCR of BKPyV may be progressively more rearranged with the progression of disease. Gosert et al. demonstrated that BKPyV with rearranged NCCR emerge in vivo in renal transplant patients and increase viral replication and cytopathology [32]. As mentioned above, archetypal forms of BKPyV are preferentially secreted in the urine but the reason remains unclear since both rearranged and archetypal sequences can be found in the BKPyV nephritis kidney. Rearranged NCCRs are more frequently observed in plasma than in urine samples and are associated with higher plasma BKPyV loads [32].

Contrary to rearranged strains, it is observed that archetypal forms of BKPyV replicate poorly in cell culture. It has thus been proposed that NCCR rearrangements are an adaptation of the virus necessary for efficient growth in diverse cell types in vitro (reviewed in [30]). In agreement with this hypothesis, Broekema et al. demonstrated that the NCCR is the major determinant of replication in cell culture [33]. This explains why most NCCRs sequenced after BKPyV amplification in cell culture contain several duplications and/or deletions [21,31] contrary to NCCRs that have been PCR amplified and sequenced directly from human urine. Importantly, limited TAg expression from the archetype NCCR production may be one key factor limiting the propagation of archetype viruses since TAg overexpression is able to rescue its replication [34].

## 4. The BKPyV Proteins

### 4.1. TAg, tAg and truncTAg

The early region of BKPyV genome encodes the TAg, tAg and truncTAg that are produced by alternatively spliced mRNAs (Figure 2). Removal of a first intron splices the first exon with the next exon, allowing translation of TAg (695 aa, 80 kDa). Alternatively, retention of this intron allows translation to reach a termination codon within the first intron, resulting in tAg (172 aa, 20 kDa). Thus, tAg shares the first 82 aa with TAg. Abend et al. also provided evidence of a third early BKPyV mRNA that encodes truncTAg (136 aa, 17 kDa) [35]. truncTAg is expressed from another alternatively spliced mRNA that is derived from the excision of a second intron from the TAg encoding mRNA. Thus, the first 133 aa of truncTAg are identical to those of TAg but the additional splice results in translation from a different reading frame, adding three new amino acids before reaching a stop codon.

TAg and truncTAg are primarily localized in the nucleus because of the presence of a nuclear localization signal (NLS) (^129^KKKRK^133^). The N-terminus of both proteins contain a J domain that shows extensive homology to the molecular chaperone proteins belonging to the DnaJ family. This domain interacts specifically with the heat shock cognate 70 (Hsc70) co-chaperone protein and is required for efficient viral replication [36,37,38]. The sequence of TAg and truncTAg also includes a conserved ^105^LXCXE^109^ motif through which both proteins associate with pRb and its family members—p107 and p130—in order to promote cell cycle entry/progression and thus viral replication [39,40]. This interaction causes displacement of E2F transcription factor family members, which subsequently activate transcription of genes involved in S phase progression and DNA synthesis [37,38].

The region that is truncated in truncTAg encompasses a DNA-binding domain (DBD), a Zinc-binding domain and an ATPase domain [37,38]. These domains confer a DNA helicase activity to TAg, which is essential for the initiation of viral genome replication. The DBD specifically recognizes the sequence GAGGC present in four copies in the origin of replication of the NCCR (Figure 2). The association of the DBD with Replication Protein A (RPA) is also essential for replication. The Zinc-binding domain is responsible for the formation of TAg hexamers that is the active helicase form. The ATPase domain provides the energy needed for helicase activity. The outside surface of the helicase domain also binds to the tumour suppressor protein p53, thus preventing cell cycle arrest and apoptosis [37,38,39,41].

In contrast to TAg and truncTAg, tAg is located in both the nucleus and the cytoplasm. This protein only shares the N-terminal J domain with TAg and truncTAg, followed by a unique region containing two zinc-fingers that inactivates protein phosphatase 2A through the formation of a complex to promote cell cycle progression [37,38].

BKPyV has been shown to cause malignant transformation of BHK21 clone 13 cells [42] and hamster kidney cells [43,44]. The cell cycle-altering functions of TAg and tAg are largely responsible for the cell transforming potential of BKPyV. The oncogenic properties of BKPyV have also been well demonstrated in experimental models [45]. Evidence in support of BKPyV as a potential cofactor in human prostate cancers has been reported [46]. Furthermore, it has recently been shown in a renal allograft tumour that BKPyV has integrated into human chromosomal DNA (but not in normal renal cells), with robust expression of TAg, disruption of late genes expression and impediment of viral replication [47]. However, based on “inadequate evidence” in humans, BKPyV is still classified as “possibly carcinogenic to humans” (Group 2B) by the WHO International Agency for Research on Cancer Working Group [48].

### 4.2. VP1, VP2 and VP3

The structural proteins VP1, VP2 and VP3 of BKPyV play important roles in the infection life cycle, in particular during the entry step and for the assembly of progeny virions. VP1 is a protein of 362 amino acids (42 kDa) that is divided into five loops, known as BC, DE, EF, GH and HI, that connect the different strands of the polypeptide [49]. As mentioned previously, VP1 forms pentamers that are present at the external portion of the viral capsid. The importance of the VP1 loops in mediating capsid assembly has been demonstrated [50]. Pentamers are built as a ring of five β-barrel-shaped VP1 monomers, tightly linked by interacting loops between the frameworks of β-strands. The N-terminal region of VP1 lies on the inside of the virion and mediates DNA binding [11]. The C-terminal subdomains of each VP1 monomer form arms that extend into neighbouring capsomers, binding them together to form virions. This is emphasized by the fact that C-terminally truncated VP1 does form capsomers that cannot assemble to form normal virus-like particles (VLPs, see Section 6) [49]. The capsid is stabilized by an extensive network of inter- and intra-pentameric disulphide bonds. VP1 also plays an essential role in viral attachment to susceptible cells. Key VP1 residues for BKPyV assembly and entry have been identified by site directed mutagenesis [50,51]. Phosphorylation of Ser-80 of VP1 has been shown to be crucial for BKPyV propagation [52]. The receptor-binding site of BKPyV has been predicted to be located in a shallow groove formed by the BC and HI loops of VP1 [4,50]. The BC loop encompasses the epitopes responsible for serotype differences between BKPyV isolates [4]. Indeed, it contains a 69 bp region, encoding aa 61–83 and named “VP1 subtyping region”, that is used to identify different serotypes [4]. Recently, we also developed a simple algorithm that enables rapid determination of the 12 BKPyV subtypes/subgroups based on a region of only 100 bp in VP1 that we called “BKPyV typing and grouping region” [53].

Each VP1 pentamer interacts with one copy of the internal protein VP2 (351 amino acids; 38 kDa) or VP3 (232 amino acids; 27 kDa) that inserts into the central cavity in a hairpin-like manner through hydrophobic interactions [11]. Both proteins are expressed from the same late mRNA transcript and share a C-terminal amino acid sequence but VP2 has a unique set of N-terminal amino acids that includes a putative myristoylation site at Gly-2. However, the modification of this site for BKPyV VP2 has not been detected by mass spectrometry [14]. The shared C-terminal segment of VP2 and VP3 contains important features, including the VP1-binding region, a DNA-binding region and a NLS [54]. These two proteins are not required for viral assembly and their removal does not affect virion stability [11,49]. In contrast, the mutation of the start codons of the minor capsid proteins alone or together reduced infectivity by over 99% compared to the WT demonstrating that these proteins are essential to create infectious virions [54]. Phosphorylation of Ser-254 of VP2 has been suggested to be critical for BKPyV propagation [52].

### 4.3. Agno

The Agno protein is a small basic protein composed of 66 amino acids (8 kDa) that is abundantly expressed in BKPyV infected cells in vitro and in vivo [55,56]. This protein localizes principally in the cytoplasm during the late phase of the BKPyV life cycle, being most intense in the perinuclear area but a small fraction can also be detected in the nucleus [55,57]. The region encompassing aa 20–42 has been shown to form an amphipathic helix that can target the protein to lipid droplets [58]. However, this region is also involved in the formation of stable dimers and oligomers whose function is unknown [59,60]. BKPyV Agno is phosphorylated with putative phosphorylation sites at Ser-7, Ser-11, Thr-21 and Ser-64 [57]. Ser-11 phosphorylation is mediated by Protein Kinase C (PKC) in cell culture [57]. In vitro studies also demonstrate that this site can be phosphorylated by PKA, PKC and PKD, while PKC and PKD can additionally phosphorylate Ser-7 and Thr-21 [57]. Ser-11 phosphorylation has an important regulative function and controls the stability of the protein as well as BKPyV propagation [57]. However, changing the Ser-11 phosphorylation site to alanine or aspartic acid does not alter co-localization with lipid droplets [58].

BKPyV with an introduced point mutation in the agno gene start codon, preventing Agno production, was found to be infectious in Vero cells, although with reduced capacity compared to wild-type BKPyV [57]. This indicates that Agno plays an important but not crucial role in BKPyV life cycle. Additionally, it has been observed that some BKPyV strains bearing a NCCR deletion including the 5′ end of the Agno coding sequence do not release infectious progeny into cell culture supernatants but produce nuclear non-infectious VLPs [61]. Furthermore, the trans-complementation of these strains with Agno rescued the production and release of infectious virions in the cell culture supernatant [61]. Thus, Agno could be involved in assembly, maturation and/or release of infectious virions. Okada et al. have shown that JCPyV Agno facilitates virion nuclear egress by destabilizing the nuclear membrane and the region of JCPyV Agno involved in this process is highly homologous to that of BKPyV Agno [62]. Indeed, the N-terminal 24 amino acids of JCPyV Agno are able to interact with Heterochromatin Protein 1α (HP1α) and to disrupt its interaction with the lamin B receptor, a transmembrane protein in the inner nuclear membrane [62]. It is thus tempting to speculate that BKPyV Agno facilitates virion nuclear egress through the destabilization of the nuclear membrane. Myhre et al. observed that the majority of lamin accumulated at the nuclear rim in BKPyV infected cells, whereas it was present throughout the nucleoplasm in uninfected cells [61]. However, no co-localization of BKPyV Agno with HP1α was detected [61].

It has been shown that Agno specifically interacts with a subset of human cellular proteins [55]. Using a yeast two-hybrid assay Johannessen et al. identified α-soluble *N*-ethylmaleimide-sensitive fusion attachment protein (α-SNAP) as a cellular partner that interacts directly with the N-terminal 38 amino acids of BKPyV Agno [63]. α-SNAP is involved in disassembly of vesicles during secretion and, through this interaction, Agno seems to influence negatively exocytosis [63]. The functional consequence of the modulation of the secretory pathway is unknown. However, this could impede antigen presentation and/or reduce the secretion of cytokines and/or interferons by infected cells thus allowing immune evasion. BKPyV Agno also interacts with proliferating cell nuclear antigen (PCNA) and inhibits DNA replication [64]. Furthermore, by analogy to JCPyV, it has been proposed that BKPyV Agno could inhibit DNA repair after DNA damage and interfere with DNA damage-induced cell cycle regulation [65]. Finally, using a bidirectional reporter vector expressing red and green fluorescent proteins under the control of BKPyV NCCR, Gosert et al. demonstrated that Agno expression negatively regulates both early and late gene expression [32]. This is in agreement with the observation that VP1 expression is increased during infection with BKPyV lacking Agno [57].

### 4.4. Putative VP4

In 2007, Daniels et al. reported that SV40 encodes a very late protein denoted VP4 whose synthesis initiates from a downstream AUG start codon within the sequence of VP2 so that VP2, VP3 and VP4 share a C-terminal sequence [66]. These authors suggested that this protein was involved in cell lysis and consequently in progeny virion release and a corresponding open reading frame is present in the BKPyV genome [66]. However, Henriksen et al. recently contradict this previous report since they failed to identify VP4 in infected cell extracts by mass spectrometry and they demonstrated that BKPyV as well as SV40 do not require VP4 for progeny release [54].

## 5. BKPyV-Encoded MicroRNAs

In 2008, Seo et al. demonstrated that like SV40, JCPyV and BKPyV encode a pre-miRNA hairpin, both arms of which are processed into two different mature miRNAs, named 5p-miRNA and 3p-miRNA (Figure 2), which are perfectly complementary to early mRNAs [67]. Importantly, they also showed that these miRNAs were expressed in individuals diagnosed with polyomavirus-associated disease. This first study suggested that both miRNAs regulate early gene expression at late times of infection through the cleavage of the early viral transcripts, which could reduce the susceptibility of infected cells to cytotoxic T cells, as described for SV40 [68]. However, it has been shown later that the miRNAs can regulate early mRNA expression before genome replication [69]. Indeed, miRNA expression is controlled by sequences in the NCCR, in a manner similar to late mRNA transcripts. Therefore, archetype virus miRNAs are robustly expressed and target early mRNAs, weakly expressed from the early promoter, for degradation. Consequently, DNA replication is limited in archetype viruses. In contrast, in rearranged variants, the miRNAs are only weakly expressed, which is not sufficient to decrease the high levels of early mRNAs that are expressed from high early promoter activity. Additionally, Bauman et al. have demonstrated that these miRNAs can target cellular genes. In particular, BKPyV-3p-miRNA and JCPyV-3p-miRNA, that are strictly identical, target the stress-induced ligand ULBP3, a protein recognized by the killer receptor NKG2D [70]. This viral miRNA-mediated ULBP3 down-regulation results in reduced NKG2D-mediated elimination of virus-infected cells by natural killer cells. This could partly explain how BKPyV escapes from the immune system and remains latent. Thus, the miRNAs may play a significant role in the mechanism of viral persistence [69].

## 6. In Vitro Model Systems for BKPyV Research

BKPyV replicates only in human or monkey cells. As mentioned above, it was first isolated after inoculation of Vero cells with urine samples [1]. For this reason, basic research studies on BKPyV are widely performed using this African green monkey kidney cell line. Other kidney cell lines of monkey or human origin are also used to study BKPyV biology (e.g., CV-1 or HEK293 cells) [55,71,72]. Primary human renal proximal tubule epithelial cells (RPTE cells) is the more suitable culture system to investigate BKPyV infection in a model that is more similar to the natural target but they are difficult to manipulate [73]. As mentioned previously, archetype forms of BKPyV replicate poorly in cell culture because of limited TAg expression. HUV-EC-C was the first cell line shown to allow propagation of archetypal BKPyV [74]. Later, the use of cell lines transformed with the SV40 TAg (e.g., 293TT and COS-7 cells that are derived from HEK293 and CV-1 cells, respectively) also permitted the propagation of archetype viruses [34]. In addition, it has been reported that BKPyV productively infects salivary gland cell lines [75]. The virus can also grow in human embryonic fibroblast cells (e.g., WI-38, HEL-299 or MRC-5 cells) that are not the normal target cells for the virus in vivo [12,72,76]. Recombinant viruses with rearranged NCCR (e.g., Dunlop, Gardner and TU strains) or archetype NCCR (e.g., WW and Dik strains) are often used [34,35] and infectious recombinant BKPyV viruses expressing reporter genes have been developed [77].

Surrogate models have also been developed. Viral capsids are formed by the assembly of VP1, VP2 and VP3 structural proteins. However, capsid-like structures can self-assemble upon overexpression of the major capsid protein VP1 alone to form VLPs resembling native virions but devoid of genetic material [11,49]. These VLPs can be generated in an array of systems including insect cells, mammalian cells, yeast or *Escherichia coli* [49]. These models provided useful information on viral morphogenesis and on the structural basis for the BKPyV antigenicity [11,20]. VLP-based vaccines that could be used before transplantation are also under development [49]. Interestingly, VLPs can encapsidate DNA in a sequence-independent manner, such as a plasmid encoding a reporter gene and deliver it into target cells [49]. One major impediment in the BKPyV field is the lack of a small animal model. The presence of a BKPyV DNA replication restriction factor in murine cell extracts has been evidenced [78].

## 7. BKPyV Entry

To successfully infect a cell, a viral particle needs to attach to the cell surface, interact with receptor(s) to become internalized and target a productive pathway. For DNA viruses, this means that the viral genome must be transported into the nucleus.

### 7.1. Attachment

BKPyV infection starts with attachment of VP1 to cellular receptors and it has been suggested that the different BKPyV subtypes/serotypes bound a distinct spectrum of cell surface receptors and thus have different cellular tropisms [6]. However, several studies suggested that polysialylated gangliosides play an important role in the initial interaction between BKPyV and target cells, as well as in BKPyV hemagglutination of human type O red blood cells [79,80,81]. Gangliosides are one type of glycosphingolipid that are enriched within the lipid raft portions of cell membranes. They consist of a ceramide and a carbohydrate moiety that is typically composed of two arms containing one or more sialic acid residues [82]. Gangliosides are usually defined by a short-hand nomenclature system in which GM, GD and GT refer to mono-, di- and trisialogangliosides, respectively, followed by a number that refers to the migration order of the gangliosides on thin-layer chromatography. Sialic acid residues are typically found in α(2,3)-linkage to galactosyl residues or in α(2,8)-linkage to other sialic acids. It has recently been demonstrated that the conserved α(2,8)-disialic acid motif on the right arm of b-series gangliosides is the minimal binding epitope for BKPyV, with the variable left arm contributing some additional contacts [51]. Thus, BKPyV is able to interact with different types of b-series gangliosides such as GD3, GD2, GD1b and GT1b [51,83]. In contrast, BKPyV virions do not interact with the monosialylated a-series gangliosides GM1 [51,83]. Furthermore, in contrast to GM1, GM2, GM3 and GD1a, the addition of GD3, GD2, GD1b and GT1b to target cells enhances the susceptibility to BKPyV infection [51,83]. It has also been shown that Abl family kinases regulate the susceptibility of cells to BKPyV infection by inhibiting host cell sialidase activity and thus modulating cell surface ganglioside levels [84].

Dugan et al. reported that enzymatic removal of α(2,3)-linked sialic acid from cells inhibited BKPyV infection and that reconstitution of asialo cells with α(2,3)-specific sialyltransferase restored susceptibility to infection [85]. In addition, they showed that inhibition of N-linked glycosylation with tunicamycin reduced infection, whereas inhibition of O-linked glycosylation did not. They thus proposed that an N-linked glycoprotein containing α(2,3)-linked sialic acid is a critical host receptor for BKPyV entry [85].

### 7.2. Internalization

Following initial attachment to the cell surface, BKPyV is internalized into the target cell. Using Cav-1 and Eps15 dominant-negative mutants that dismantle caveolin and clathrin-dependent endocytosis respectively, Eash et al. demonstrated that BKPyV entry into Vero cells is independent of clathrin-coated-pit assembly but reliant on cholesterol-dependent, caveola-mediated endocytosis [86]. Similar results were obtained by Moriyama et al. in RPTE cells [87,88]. Kinetic studies showed that the majority of BKPyV virions reach a neutralizing antibody-resistant compartment between 2 and 4 h after initiation of infection [86] and that colocalization of BKPyV with Cav-1 peaked after 4 h [87]. However, additional studies are required to further define the BKPyV endocytosis process since Zhao et al. recently obtained discrepant results in RPTE cells and observed that BKPyV entered cells via a caveolin- and clathrin-independent pathway [89].

### 7.3. Trafficking through the Endoplasmic Reticulum

After endocytosis, BKPyV may enter the endosomes [90]. In addition, based on transmission electron microscopy of infected cells, it has been observed that BKPyV virions traffic to smooth tubular structures contiguous with rough endoplasmic reticulum (ER) [91]. The intracellular trafficking of BKPyV in Vero and RPTE cells was reported to be sensitive to nocodazole and thus to rely on an intact microtubule network [92,93,94], while an intact actin cytoskeleton is not required for BKPyV intracellular transport [92]. In both cell types, trafficking of BKPyV is dynein-independent. In contrast, the use of paclitaxel to study the importance of microtubule dynamics in the intracellular trafficking of BKPyV gave rise to conflicting results depending on cells used for infection. BKPyV movement was dependent upon microtubule dynamics in RPTE cells [93], which was not the case in Vero cells [92].

The use of lysosomotropic agents that disrupt the acidification of intracellular organelles such as chloroquine and ammonium chloride (NH_4_Cl) also demonstrated that BKPyV entry involves a pH-dependent step during the first 2 h, suggesting that acidification and maturation of the endosomes are essential for BKPyV infection [86,94]. BKPyV entry is sensitive to brefeldin A and Retro-2cycl, two drugs that block the retrograde transport to the ER but colocalization of BKPyV with markers of the Golgi apparatus has never been observed [83,93,94,95]. It is thus likely that BKPyV virions use a pathway that does not involve the Golgi apparatus or that they pass too rapidly through the Golgi apparatus to be detected. However, the absence of infection inhibition using dynein complex inhibitor, that also disrupts the Golgi apparatus morphology, supports the idea that the Golgi apparatus is bypassed [87]. The kinetics of inhibition by nocodazole and BFA treatments are similar, suggesting that the virus moves along microtubules to reach the ER [94]. By implementing a whole human genome small interfering RNA (siRNA) screen, Zhao et al. recently identified key components involved in BKPyV late-endosome-to-ER trafficking. They showed that, after endosomal sorting, BKPyV travels in Rab18-positive vesicles that are captured by the NRZ complex, a structure formed by NAG, RINT1 and ZW10 proteins, involved in retrograde transport to the ER. They also evidenced that syntaxin 18 mediates vesicle fusion with the ER membrane [90]. Studies using brefeldin A and colocalization with markers of the ER indicate that BKPyV reaches this compartment at ~10 h post-entry [93,94]. Interestingly, autophagy may play a role in BKPyV entry and/or intracellular trafficking since infection is sensitive to autophagy inhibitors during the first 8 hours and decreased in cells under-expressing autophagy genes such as ATG7 and Beclin-1 [96]. Data also suggested that early steps during BKPyV entry are sensed by the Akt-mTOR pathway [97].

### 7.4. Release from the ER and Nuclear Entry

A striking feature of polyomaviruses is their ability to traffic through the ER before reaching the cytosol during the entry process. Importantly, it has been observed that disruption of trafficking to the ER influences disulphide bond isomerization and the cleavage pattern of the major capsid protein VP1 [94]. Furthermore, ERdj5 has been identified as an ER reductase that cooperates with Protein Disulphide Isomerase to reduce BKPyV disulphide bonds and thus promote BKPyV infection [98]. It is thus likely that trafficking through the ER permits BKPyV to benefit from chaperones, disulphide isomerases and reductases to facilitate the capsid uncoating process and mediate viral ER-to-cytosol transport. Several host factors involved in the ER-associated protein degradation (ERAD) pathway and the proteasome have been implicated in BKPyV entry into the cytosol from the ER (for review see [99]). The ERAD machinery is normally required for retro-translocation of certain misfolded proteins from the ER for proteasomal degradation and involves several proteins of the Hsp70, Hsp110, membrane J-protein or Derlin families. ERAD and proteasome inhibition, with Eeyarestatin I and epoxomicin, respectively, causes an accumulation of partially uncoated virions with exposed VP2/VP3 in the ER and inhibits BKPyV infection [71]. However, proteasome and ERAD inhibitors do not prevent entry of VP1 into the cytosol from the ER [71]. Indeed, it has been shown that VP2 is exposed in the ER and it is likely that partial uncoating creates a hydrophobic surface exposing VP2/VP3 that binds to and integrates into the ER membrane, leading to the release of the partially uncoated virus into the cytosol [71,95]. Prior to membrane penetration, the hydrophobic BKPyV may be maintained in a soluble state by recruiting the ER-resident Hsp70 BiP [100,101]. The Nuclear Exchange Factor activity of Grp170 may trigger the release of ER-localized BKPyV from BiP by converting ADP-BiP to ATP-BiP [100]. Membrane-embedded BKPyV may induce the lateral reorganization of different ER membrane proteins such as the J-proteins B12, B14 and to a lesser extent, B11 and C18 [101]. The ER membrane component Derlin-1 has also been reported to interact with VP1 and mediate ER-to-cytosol transport of BKPyV but its precise molecular contribution to the membrane penetration event is unclear [94]. Then, a cytosolic complex containing Hsp105 and SGTA proteins may be recruited to extract BKPyV to the cytosol [102,103].

Once in the cytosol, the viral genome has to be transported into the nucleus before it can be replicated. Bennett et al. provided evidence that after the viral particle exits the ER, NLS of VP2 and VP3 are exposed and used by the importin α/β1 import pathway to bring the viral genome into the nucleus via the nuclear pore complex [104]. However, an alternative nuclear entry pathway existing along with import through the nuclear pore has also been suggested [104].

## 8. BKPyV Gene Expression and Genome Replication

The mechanisms of BKPyV replication have largely been extrapolated from work on SV40, a closely related polyomavirus. As soon as the genome enters the nucleus, early viral genes are transcribed and early viral proteins are translated in the cytoplasm. TAg is then translocated into the nucleus thanks to the NLS. By binding to the pRb and p53 tumour suppressors, TAg stimulates cell cycle progression and counteracts apoptosis, thereby providing the host DNA synthetic machinery that is necessary for viral genome replication. Data also show that conditions that promote cell growth and division, for instance through MAP kinase or Akt/mTOR signaling pathways, increase BKPyV replication in vitro [76,105]. In addition, tAg contributes to BKPyV replication by orchestrating cell cycle progression through inhibiting the enzymatic activity of protein phosphatase 2A. BKPyV does not encode a DNA polymerase and requires only one viral protein for viral genome replication, the multifunctional TAg. All other replication factors are supplied by the host cell. TAg initiates replication of the viral DNA by binding to GAGGC motifs in the origin of replication and forming two hexamers exhibiting helicase activity, in a head-to-head orientation. In an ATP-dependent process, the TAg double hexamer locally unwinds the double stranded DNA, in a bidirectional manner. Then, TAg recruits RPA, which covers the resulting stretches of single stranded-DNA as well as topoisomerase I that facilitates unwinding. In the following step, DNA polymerase α-primase (Pol α-primase) is recruited. It synthesizes short RNA primers that are elongated by the DNA polymerase function of the enzyme complex. Leading and lagging strand synthesis is then completed by DNA polymerase δ and Pol α-primase with the help of RPA, proliferating cell nuclear antigen (PCNA) and replication factor C [106,107].

Transcriptomic analyses demonstrated that BKPyV deregulates the expression of numerous cellular genes [108,109]. It has been shown that viral DNA replication activates the cellular DNA damage response through ATM and ATR to protect cells from BKPyV-induced host DNA damage [110,111]. In the absence of either kinase, severe DNA damage accumulated during BKPyV infection. BKPyV DNA replication also causes a dramatic reorganization of Promyelocytic Leukemia Nuclear Bodies (PML-NBs), with a decrease in PML-NBs number and an increase in size [112]. Interestingly, BKPyV DNA foci were found adjacent to PML-NBs during late infection and it has been suggested that the reorganization of PML-NBs could be a strategy used by BKPyV to inactivate their intrinsic antiviral functions. Late proteins expression is detected during late infection. It is permitted by two synergistic mechanisms governed by TAg, the increase of DNA templates and the activation of transcription from the late promoter.

## 9. BKPyV Assembly and Release

After translation in the cytoplasm, the viral capsid proteins VP1, VP2 and VP3 are imported into the nucleus for virion assembly. It has been suggested that the minor capsid proteins NLS were not involved in this transport and that only the VP1 NLS was necessary to import VP1–VP2 and VP1–VP3 complexes in the nucleus [104]. Association of VP1 with chaperone proteins in the cytoplasm may prevent premature assembly of virions [49]. In the nucleus, the high calcium concentration may enable the assembly of viral capsomers around newly synthesized genomes [49]. Progeny virions start to appear in the nucleus of infected cells at 2 days post-infection [73]. A mean of 6000 BKPyV virions per infected cell has been estimated in renal biopsies with polyomavirus-associated nephropathy [8]. As a result, dense crystal-like arrays of particles corresponding to nuclear inclusion structures can be seen by electron microscopy [91], as typically observed in “decoy cells” that are exfoliated into the urine.

While many studies have investigated the mechanism of entry and assembly of BKPyV, little is known about the release of newly formed virions from infected cells. It is generally assumed that non-enveloped viruses are released through passive means such as host cell lysis and it has been shown that BKPyV undergoes a lytic replication cycle in RPTE cells [73]. However, strong cytopathic effects are rarely seen in BKPyV-infected cells as compared to SV40-infected cells [54]. Furthermore, it remains questionable whether this holds true in vivo in the presence of an intact immune system, since the virus establishes lifelong persistent infections. Besides, it is interesting to note that a non-lytic egress of BKPyV from RPTE cells has recently been reported [113]. These authors demonstrated that approximately 1% of total infectious virus progeny is released into the extracellular environment through a route that can be inhibited by 4,4′-diisothiocyano-2,2′-stilbenedisulfonic acid (DIDS), an anion channel blocker known to affect cellular secretion pathways. Additionally, they observed that, in treated cells, BKPyV virions become trapped in acidic compartments of lysosomal or late endosomal origin. However, it remains to be determined how BKPyV virions traffic from the nucleus to the lumen of these compartments. As mentioned above, Agno could be involved in assembly, maturation and/or release of infectious BKPyV virions. However, further studies are required to decipher the contribution of this protein in lytic and/or non-lytic egress of BKPyV.

## 10. Concluding Remarks and Future Directions

**Figure 3:**
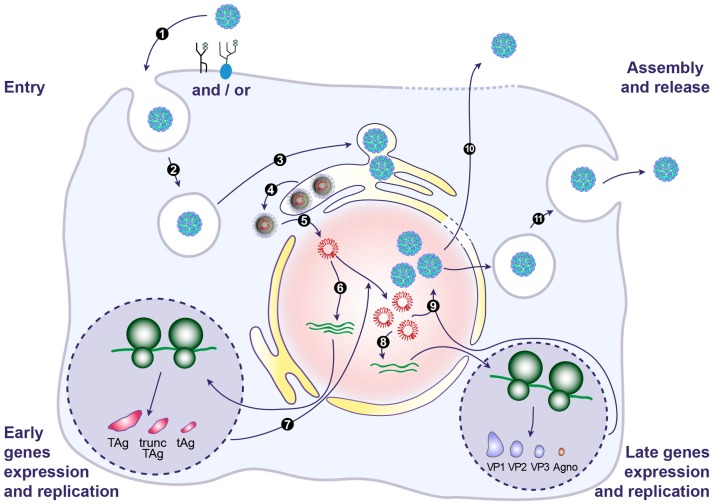
Model of the BKPyV life cycle. BKPyV infection begins with binding of virions to the ganglioside receptors (particularly GT1b and GD1b) and/or an N-linked glycoprotein containing α(2,3)-linked sialic acid, at the cell surface (1). This is followed by internalization potentially through a caveola-mediated endocytosis step within the first 4 h after adsorption (2). The virus subsequently traffics from the late endosomes to the endoplasmic reticulum (ER), where it arrives approximately 10 h post-infection (3). In the ER, virions benefit from chaperones, disulphide isomerases and reductases to facilitate the partial capsid uncoating. This creates a hydrophobic surface exposing VP2/VP3 that binds to and integrates into the ER membrane, leading to the release of partially uncoated viruses into the cytosol, a process that also involves the ER-associated protein degradation (ERAD) machinery (4). The viral genome is then transported into the nucleus via the nuclear pore complex thanks to VP2/VP3 NLS and the importin α/β1 import pathway (5). Expression of early genes occurs approximately 24 h post-infection (6). Early proteins are translocated into the nucleus where they serve to initiate viral DNA replication (7). Late genes are then expressed (8). VP1, VP2 and VP3 are translocated into the nucleus where they self-assemble to form capsids into which newly synthetized double stranded viral DNA is packaged (9). Progeny virions are mainly released from infected cells after cell lysis (10). However, a small fraction of progeny virions may also be released into the extracellular environment through a non-lytic egress that depends on the cellular secretion pathway (11).

With implementation of more potent immunosuppressive drugs in the last decade, BKPyV has become an emerging pathogen, particularly in kidney and bone marrow transplant recipients. In this review, we provide an update on recent advances in understanding the biology of BKPyV. Based on these data, we present a model of the BKPyV life cycle in Figure 3. BKPyV principally hijacks the host cell DNA replication machinery for its own reproduction. This partly explains the absence of approved specific antiviral and the lack of effective treatment. Additional research needs to be done to better understand BKPyV biology and to enable the identification of druggable targets to block BKPyV replication. The development of an efficient and robust high-throughput cellular screening assay would be useful to find potential drug candidates. In addition, establishing an appropriate animal model of the BKPyV infection would be a major milestone. Finally, progress in the field of the immune response directed against BKPyV is needed to aid the design of a vaccine.
